# Supplementary material for: Sensory experience steers representational drift in mouse visual cortex
Source: Nat Commun. 2024 Oct 23;15:9153. doi: 10.1038/s41467-024-53326-x (PMC11499870; doi:10.1038/s41467-024-53326-x)
Supplement: Supplementary file 1 — Supplementary Information [file 41467_2024_53326_MOESM1_ESM.pdf]

Supplementary information for:

Sensory experience steers representational drift in mouse visual cortex

Bauer*, Lewin* et al.

Supplementary Figures

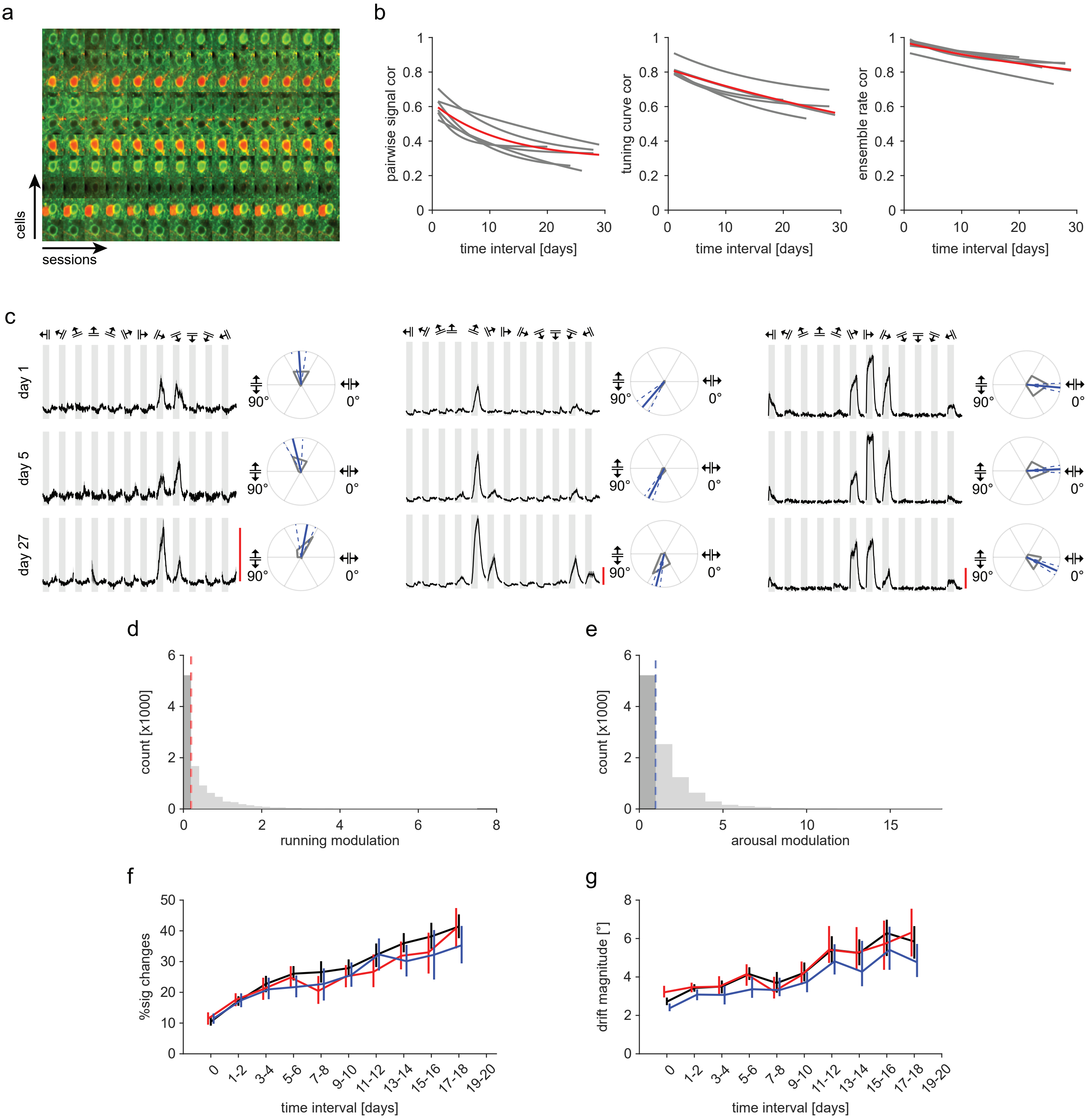
a
cells
sessions
b
pairwise signal cor
tuning curve cor
ensemble rate cor
time interval [days]
c
day 1
day 5
day 27
90°
0°
d
count [x1000]
running modulation
e
count [x1000]
arousal modulation
f
%sig changes
time interval [days]
g
drift magnitude [°]
time interval [days]

Supplementary Figure 1

a Example neurons tracked across all sessions. Image crops are 32 μm.

b Correlation decay fits of pairwise signal correlation, tuning curve correlation and ensemble rate correlation. Decay fits for all six mice in gray, average decay fits in red. PSC decay fit: $y = 0.29 + 0.33e^{-0.083*x}$; TCC decay fit: $y = 0.0039 + 0.81e^{-0.013*x}$; ERC decay fit: $y = 0.69 + 0.28e^{-0.029*x}$.

c Responses of three example neurons from the same mouse on three days. Gray bars: stimulus window of 5 s, grating directions indicated above. Average stimulus response traces in black, with S.E.M. in gray. Scale bar: 100 ΔF/F. Right, corresponding polar plots, with gray lines indicating mean response. PO in blue with 95% confidence intervals as blue dashed lines.

d Histogram of estimated running modulation for all PO changes. Running modulation: correlation between running and cell activity, multiplied by change in running speed across sessions. Median 0.20 in red (n = 10441 comparisons from 6 mice).

e As in **d** but for arousal modulation (derived from pupil diameter). Median 0.99 in blue (n = 10441 comparisons from six mice).

f Percent concurrently tuned cells that significantly changed their PO vs. time interval (black). N = 6 mice. To test if behavioral changes across sessions cause PO drift, we excluded the 50% of PO changes that were most influenced by changes in running speed (red; left of red dashed line in **d**) or arousal (blue; left of blue dashed line in **e**). Error bars are bootstrapped 95% confidence intervals.

g Same as **f,** but for PO drift magnitude vs time interval. N = 6 mice. Error bars are bootstrapped 95% confidence intervals.

Source data are provided as a Source Data file.

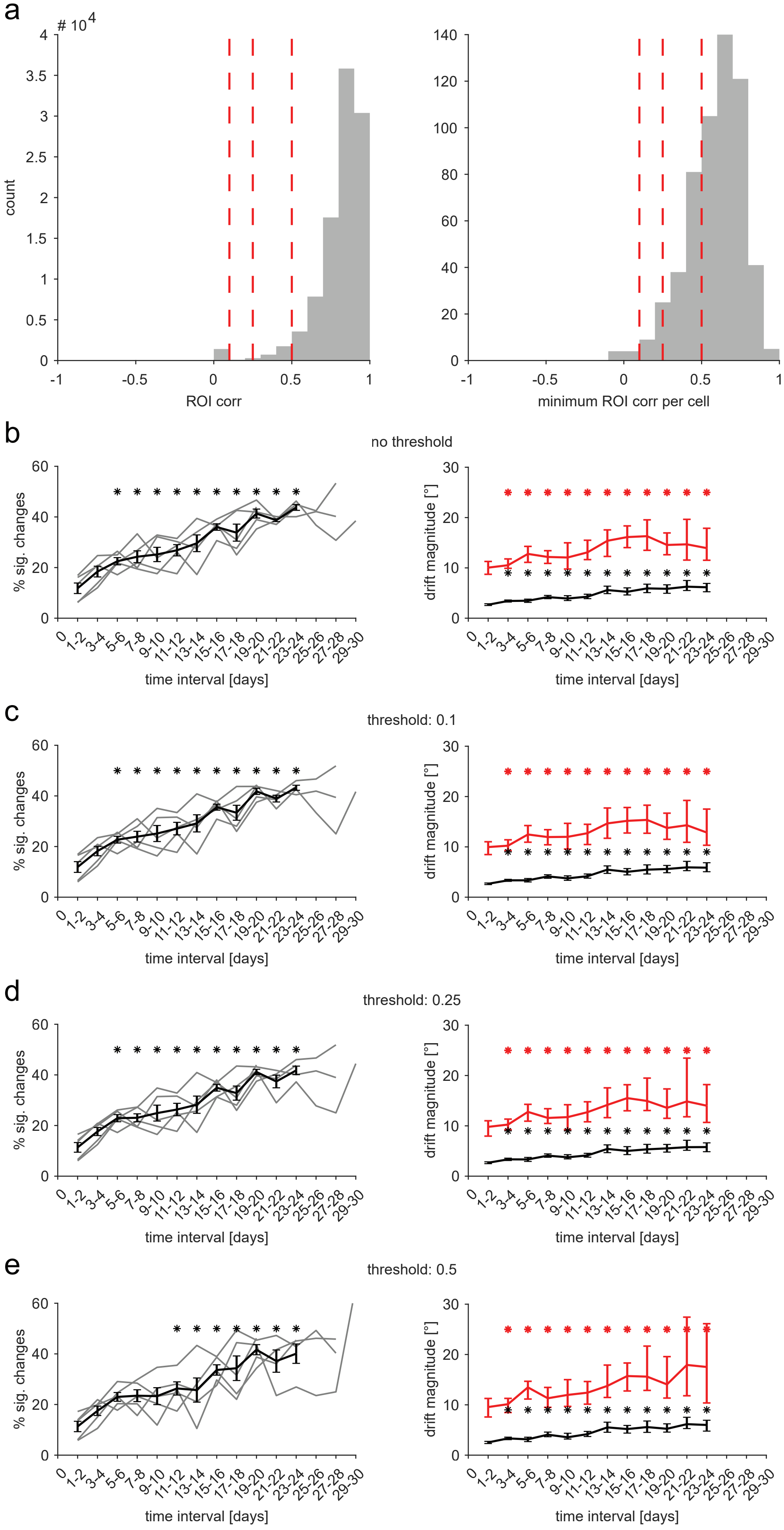
a
10^4
count
ROI corr
minimum ROI corr per cell
b
no threshold
% sig. changes
drift magnitude [°]
time interval [days]
c
threshold: 0.1
d
threshold: 0.25
e
threshold: 0.5

Supplementary Figure 2

a Left: Distribution of pixel-wise correlations between cropped areas around region of interest (ROI; as depicted in Supplementary Fig. 1a) for all pairs of matched ROIs. Right: Lowest ROI correlation for each neuron across all time intervals. Red dashed lines indicate cutoff thresholds of 0.1, 0.25 and 0.5.

b Fraction of significant PO changes (left) and median absolute PO changes (drift magnitude; right) for all PO changes in black and only significant PO changes in red (573 neurons from five mice).

c Same as **b**, but excluding neurons with a minimum ROI correlation below 0.1 (8 of 573 neurons excluded).

d Same as **b**, but excluding neurons with a minimum ROI correlation below 0.25 (25 of 573 neurons excluded).

e Same as **b**, but excluding neurons with a minimum ROI correlation below 0.5 (161 of 573 neurons excluded).

Source data are provided as a Source Data file.

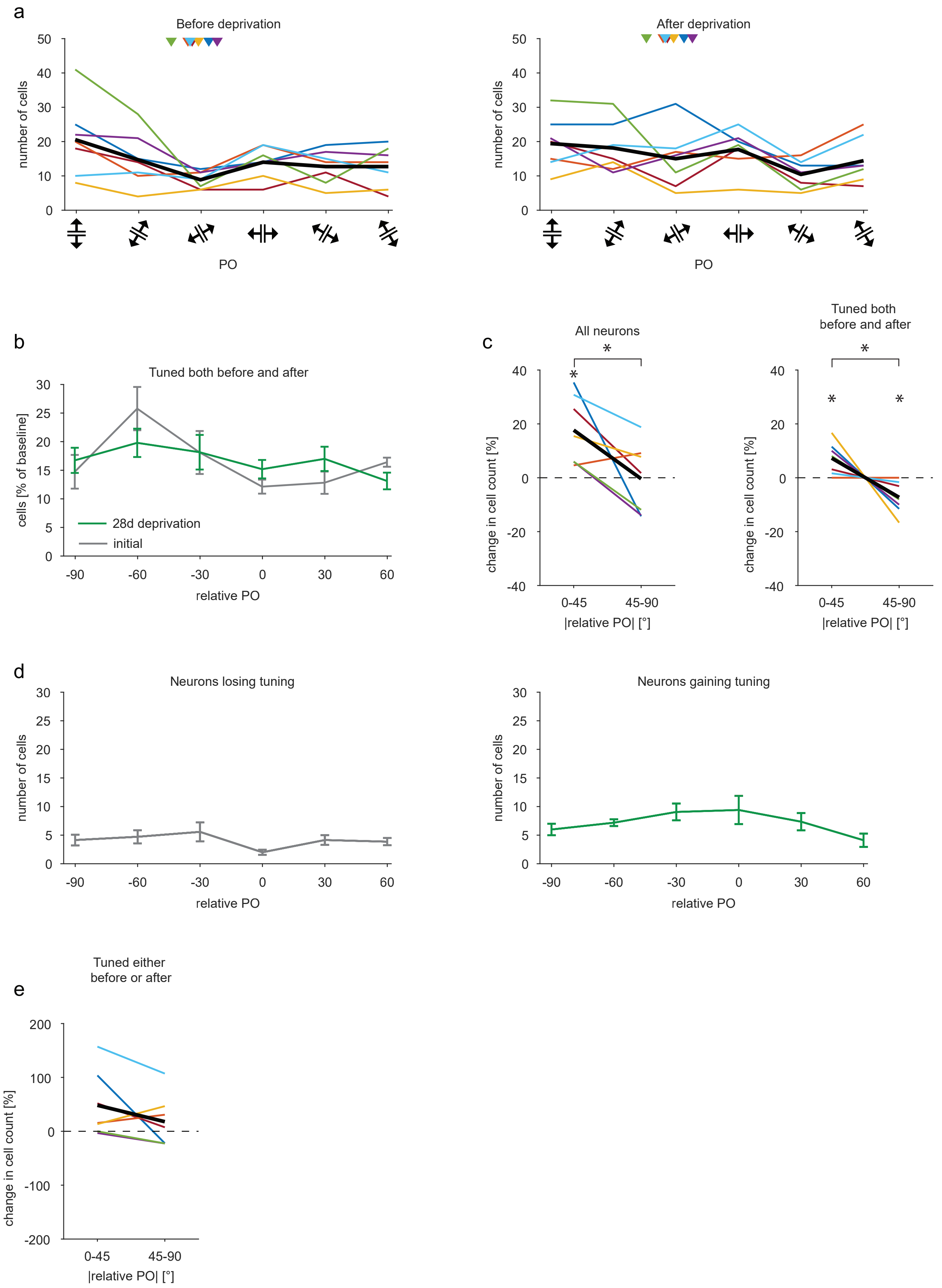
a
Before deprivation
After deprivation
number of cells
50
40
30
20
10
0
PO
b
Tuned both before and after
cells [% of baseline]
28d deprivation
initial
-90
-60
-30
0
30
60
relative PO
c
All neurons
Tuned both
before and after
*
change in cell count [%]
0-45
45-90
|relative PO| [°]
d
Neurons losing tuning
Neurons gaining tuning
number of cells
relative PO
e
Tuned either
before or after
change in cell count [%]
200
100
0
-100
-200
0-45
45-90
|relative PO| [°]

Supplementary Figure 3

a Distribution of true POs (relative to head angle) before (left) and after (right) deprivation for each mouse (colored lines) and on average (black line). Experienced orientation through the cylinder lens goggles for each mouse is indicated by arrow heads. n = 835 neurons from seven mice. Color code for individual mice matched across **a**, **c**, and **f**.

b Distribution of PO relative to the experienced orientation (0°) as a percentage relative to the initial total cell count, only including neurons that were orientation-tuned on both sessions. rPO distribution prior to and after deprivation, in gray and green respectively. n = 412 neurons from seven mice. Plotted are means across mice with error bars as S.E.M.

c Statistical analysis of population effect of orientation deprivation. Compared are the relative cell number changes, within two PO bins (bins are relative to the experienced orientation). Colored lines are seven individual mice, black lines are means. One sample T-tests were used to compare within bin changes, (all neurons: $t(6)3.62$, $p = .011$ and $t(6)\text{-}0.0827$, $p = .937$, n = 835; only neurons tuned before and after deprivation: $t(6)3.21$, $p = .018$ and $t(6)\text{-}3.21$, $p = .018$, n = 412). Stars indicate p values below a Bonferroni corrected alpha of 0.05. Paired T-tests were used for difference across bins (all neurons: $t(6)2.85$, $p = .029$; only neurons tuned before and after deprivation: $t(6)3.21$, $p = .018$). Stars indicate significance at $p < .05$.

d Distribution of neurons lost (left) and gained (right) across POs relative to the experienced orientation (0°). Left: only neurons that were tuned before and untuned after deprivation. 171 neurons (left) and 252 neurons (right) from seven animals. Plotted are means across mice with error bars as S.E.M.

e Same as **d** but for change in cell number only including neurons that were tuned either before or after deprivation but not both. One sample T-tests for comparison within PO bin $t(6)2.1$, $p = .080$ and $t(6)0.977$, $p = .367$. Paired T-test for difference across bins $t(6)1.56$, $p = .170$.

Source data are provided as a Source Data file.

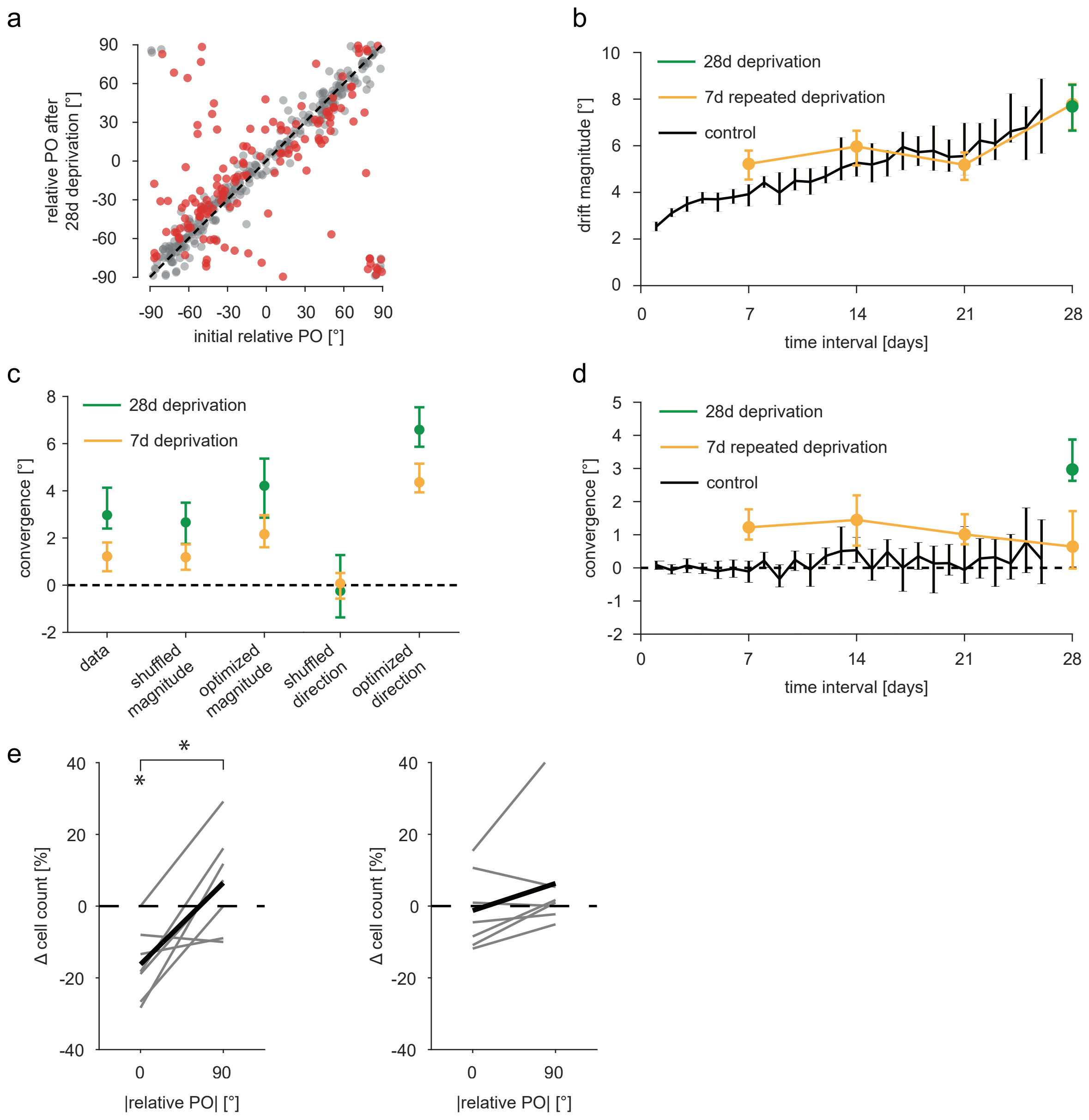
a
relative PO after 28d deprivation [°]
initial relative PO [°]
b
28d deprivation
7d repeated deprivation
control
drift magnitude [°]
time interval [days]
c
28d deprivation
7d deprivation
convergence [°]
data
shuffled magnitude
optimized magnitude
shuffled direction
optimized direction
d
28d deprivation
7d repeated deprivation
control
convergence [°]
time interval [days]
e
Δ cell count [%]
|relative PO| [°]
*
*
Δ cell count [%]
|relative PO| [°]

Supplementary Figure 4

a rPO before and after orientation deprivation for neurons that were orientation-tuned on both imaging session. N = 7 mice. Red: significant changes, gray: non-significant changes.

b Median drift magnitude (|ΔPO|) for different time intervals under baseline visual conditions (black, six mice) and orientation deprived visual conditions (orange, eight mice deprived for seven days repeatedly; green seven mice deprived for 28 days).

c Median convergence (Δ|rPO|) before and after shuffling or optimizing drift magnitudes or direction. Optimizing magnitude refers to ordering the magnitudes across neurons such that the further away from the experienced orientation a neuron is the larger its drift magnitude, while retaining the original direction of PO change for each neuron. Optimization of drift direction refers to assigning each neuron a PO change such that it reduces the difference to the experienced orientation, while keeping drift magnitude the same, i.e. all neurons drift toward the experienced orientation. These optimizations give estimates of the upper bound to PO convergence for the two hypotheses in **Fig. 2d and e** given unchanged overall drift magnitudes.

d Median convergence (Δ|rPO|) for different time intervals under baseline visual conditions (black, six mice) and orientation deprived visual conditions (orange, eight mice deprived for seven days repeatedly, i.e. interrupted orientation deprivation; green seven mice deprived for 28 days). Positive Δ|rPO| indicate changes towards the experienced orientation.

e Statistical analysis of population level of recovery (left) and across the complete experiment (right). Compared are the relative cell number changes, within two PO bins (bins are relative to the experienced orientation). Gray lines are seven individual mice, black lines are means. One sample T-tests were used to compare within bin changes, (from left to right: $t(6)$-4.27, $p = .005$ and $t(6)$1.22, $p = .269$; $t(6)$-0.309, $p = .768$ and $t(6)$0.998, $p = .357$). Stars indicate p values below a Bonferroni corrected alpha of 0.05. Paired T-tests were used for difference across bins (from left to right: $t(6)$-3.86, $p = .008$; $t(6)$-1.83, $p = .117$). Stars indicate significance at $p < .05$.

Source data are provided as a Source Data file.

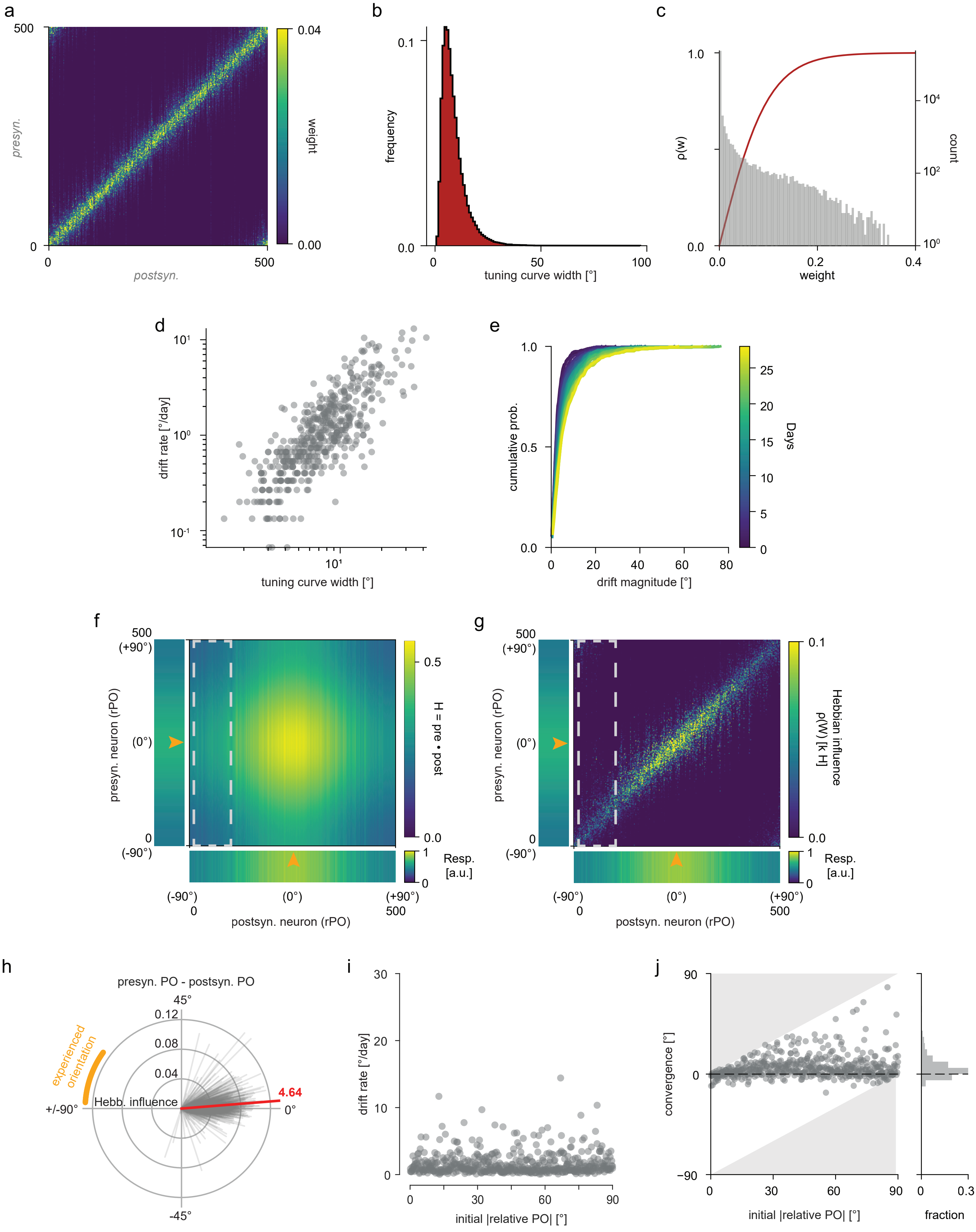

a
500
0
presyn.
postsyn.
0.04
0.00
weight
b
0.1
0.0
frequency
0
50
100
tuning curve width [°]
c
1.0
0.0
ρ(w)
0.0
0.2
0.4
weight
count
d
drift rate [°/day]
tuning curve width [°]
e
1.0
0.5
0.0
cumulative prob.
0
20
40
60
80
drift magnitude [°]
Days
25
20
15
10
5
0
f
presyn. neuron (rPO)
500
(+90°)
(0°)
0
(-90°)
H = pre • post
0.5
0.0
Resp.
[a.u.]
postsyn. neuron (rPO)
(-90°)
(0°)
(+90°)
g
Hebbian influence
ρ(W) [k H]
0.1
0.0
h
presyn. PO - postsyn. PO
45°
0.12
0.08
0.04
experienced
orientation
Hebb. influence
+/-90°
0°
-45°
4.64
i
30
20
10
0
drift rate [°/day]
0
30
60
90
initial |relative PO| [°]
j
90
0
−90
convergence [°]
initial |relative PO| [°]
0
0.3
fraction

Supplementary Figure 5

a Initial synaptic connectivity matrix, with each postsynaptic neuron receiving weighted synaptic input from corresponding presynaptic neurons with strengths following a Gaussian-shaped profile. This sets up an initial feedforward mapping with postsynaptic neurons combining the preferred orientation of their inputs. The Gaussian-shaped input weight profiles vary in width, resulting in correspondingly varying tuning width of the post synaptic neurons.

b Initial tuning curve widths are chosen to be lognormally distributed across the population of postsynaptic neurons. Parameters of the lognormal distribution are chosen such that the resulting distribution of drift magnitudes match the data ($\mu = 2, \sigma = 0.6$).

c Histogram of weights after 28 days of baseline conditions in gray (right y-axis), overlaid with the propensity function $\rho(w)$ in red (left y-axis).

d Initial tuning curve widths are correlated with the rate at which neurons drift, with wider weight profiles leading to a faster drift rate.

e Cumulative probability distribution of the absolute size of PO changes at different intervals in the model under baseline conditions, similarly plotted as for experimental data in **Fig. 1f**.

f Mean Hebbian component H of synaptic weight changes accumulated over the 28-day deprivation period, calculated as the outer product of presynaptic and postsynaptic neuronal activities during this time window. Left and bottom bars: mean presynaptic and postsynaptic activity during the deprivation period. Pre- and postsynaptic neurons are sorted by their initial PO. Orange arrow heads indicate experienced orientation. Gray dashed box indicates synapses plotted in h.

g Similar to f but showing mean Hebbian influence on synaptic weight changes accumulated over the 28-day deprivation period, given by the Hebbian component weighted by the Hebbian strength k and the propensity function: $\rho(w) * [kH]$. Gray dashed box indicates synapses plotted in h.

h Hebbian influence for synapses between presynaptic neurons and postsynaptic neurons initially tuned far from the experienced orientation (gray box in f and g). Angle indicates PO difference between presynaptic and postsynaptic neuron. Synapses with a positive difference are closer to the experienced orientation than those with negative values. The distribution is biased towards synapses with presynaptic neurons tuned closer to the experienced orientation (circular mean: 4.64°). This allows postsynaptic neurons with POs far from the experienced orientations to progressively drift towards the experienced orientation.

i Drift rate under deprivation conditions as a function of a model neuron's initial |rPO|. The drift rate of a neuron is independent of its initial distance from the experienced orientation.

j Convergence (absolute change in |rPO|) as a function of a model neuron's initial |rPO| under deprivation conditions, as shown for the data in **Fig. 2h**. Data are projected as a histogram onto the y-axis: the mean convergence value is positive, indicating that on average neurons undergo drift in the direction of the experienced orientation.

Source data are provided as a Source Data file.

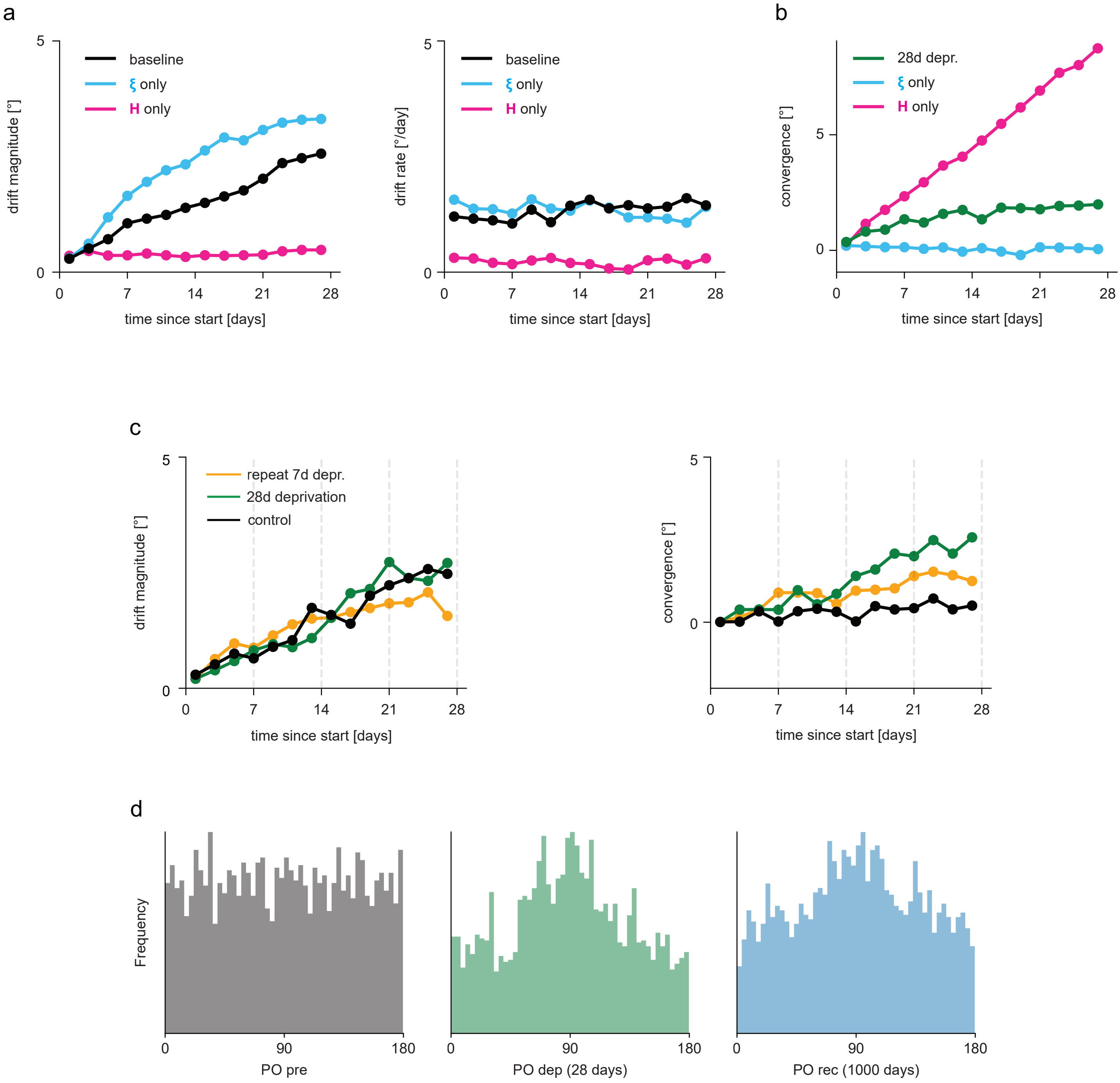
a
drift magnitude [°]
baseline
ξ only
H only
time since start [days]
drift rate [°/day]
baseline
ξ only
H only
time since start [days]
b
convergence [°]
28d depr.
ξ only
H only
time since start [days]
c
drift magnitude [°]
repeat 7d depr.
28d deprivation
control
time since start [days]
convergence [°]
time since start [days]
d
Frequency
PO pre
PO dep (28 days)
PO rec (1000 days)

Supplementary Figure 6

a Effects of omitting either Hebbian plasticity or synaptic volatility under baseline conditions on drift magnitude (left) and drift rate (right).

b Effects of omitting either Hebbian plasticity or synaptic volatility under deprivation conditions on convergence.

c Impact of interrupting the deprivation period every 7 days (dashed gray lines) with full range of orientation stimuli. Drift magnitude is unaffected while convergence towards the experienced orientation is reduced compared to uninterrupted stimulus deprivation.

d Distributions of POs at initialization, after 28 days of deprivation and after 1000 days of recovery. Data are pooled over 10 model iterations. The latter two are both significantly different from a uniform distribution (one-sample Kolmogorov-Smirnov test: $D = 0.008$, $p = .994$ (left), $D = 0.072$, $p < .005$ (center), $D = 0.053$, $p < .005$ (right)).

Source data are provided as a Source Data file.
