# Supplementary material for: Sensory experience steers representational drift in mouse visual cortex
Source: Nat Commun. 2024 Oct 23;15:9153. doi: 10.1038/s41467-024-53326-x (PMC11499870; doi:10.1038/s41467-024-53326-x)
Supplement: Supplementary file 3 — Reporting Summary [file 41467_2024_53326_MOESM3_ESM.pdf]

nature portfolio

Corresponding author(s): Joel Bauer, Mark Hübener

Last updated by author(s): Sep 13, 2024

Reporting Summary

Nature Portfolio wishes to improve the reproducibility of the work that we publish. This form provides structure for consistency and transparency in reporting. For further information on Nature Portfolio policies, see our Editorial Policies and the Editorial Policy Checklist.

Statistics

For all statistical analyses, confirm that the following items are present in the figure legend, table legend, main text, or Methods section.

n/a	Confirmed	
☐	☒	The exact sample size (n) for each experimental group/condition, given as a discrete number and unit of measurement
☐	☒	A statement on whether measurements were taken from distinct samples or whether the same sample was measured repeatedly
☐	☒	The statistical test(s) used AND whether they are one- or two-sided<br>*Only common tests should be described solely by name; describe more complex techniques in the Methods section.*
☐	☒	A description of all covariates tested
☐	☒	A description of any assumptions or corrections, such as tests of normality and adjustment for multiple comparisons
☐	☒	A full description of the statistical parameters including central tendency (e.g. means) or other basic estimates (e.g. regression coefficient) AND variation (e.g. standard deviation) or associated estimates of uncertainty (e.g. confidence intervals)
☐	☒	For null hypothesis testing, the test statistic (e.g. F, t, r) with confidence intervals, effect sizes, degrees of freedom and P value noted<br>*Give P values as exact values whenever suitable.*
☒	☐	For Bayesian analysis, information on the choice of priors and Markov chain Monte Carlo settings
☒	☐	For hierarchical and complex designs, identification of the appropriate level for tests and full reporting of outcomes
☐	☒	Estimates of effect sizes (e.g. Cohen's d, Pearson's r), indicating how they were calculated

Our web collection on statistics for biologists contains articles on many of the points above.

Software and code

Policy information about availability of computer code

Data collection	Data were collected using Matlab versions 2014 to 2017 and LabView versions 2012 and 2016. The software 'Scanimage version 4' (Vidrio Technologies) was used to acquire two-photon microscopy data. Visual stimuli were presented using Psychophysics Toolbox extensions (version 3).
Data analysis	Data analysis was done using Matlab version 2016-2020 and Python version 3.8 (using Suite2P version v0.10.4; DeepLabCut v2.2.3). Code will be available on GitHub: https://github.com/Joel-Bauer/orientation_tuning_drift and https://github.com/betsyherbert/bauer-lewin-drift-2024/tree/main

For manuscripts utilizing custom algorithms or software that are central to the research but not yet described in published literature, software must be made available to editors and reviewers. We strongly encourage code deposition in a community repository (e.g. GitHub). See the Nature Portfolio guidelines for submitting code & software for further information.

Data

Policy information about availability of data

All manuscripts must include a data availability statement. This statement should provide the following information, where applicable:

- Accession codes, unique identifiers, or web links for publicly available datasets
- A description of any restrictions on data availability
- For clinical datasets or third party data, please ensure that the statement adheres to our policy

Data will be made available at https://gin.g-node.org/Joel-Bauer/orientation_tuning_drift

Research involving human participants, their data, or biological material

Policy information about studies with human participants or human data. See also policy information about sex, gender (identity/presentation), and sexual orientation and race, ethnicity and racism.

Reporting on sex and gender	*Use the terms sex (biological attribute) and gender (shaped by social and cultural circumstances) carefully in order to avoid confusing both terms. Indicate if findings apply to only one sex or gender; describe whether sex and gender were considered in study design; whether sex and/or gender was determined based on self-reporting or assigned and methods used. Provide in the source data disaggregated sex and gender data, where this information has been collected, and if consent has been obtained for sharing of individual-level data; provide overall numbers in this Reporting Summary. Please state if this information has not been collected. Report sex- and gender-based analyses where performed, justify reasons for lack of sex- and gender-based analysis.*
Reporting on race, ethnicity, or other socially relevant groupings	*Please specify the socially constructed or socially relevant categorization variable(s) used in your manuscript and explain why they were used. Please note that such variables should not be used as proxies for other socially constructed/relevant variables (for example, race or ethnicity should not be used as a proxy for socioeconomic status). Provide clear definitions of the relevant terms used, how they were provided (by the participants/respondents, the researchers, or third parties), and the method(s) used to classify people into the different categories (e.g. self-report, census or administrative data, social media data, etc.) Please provide details about how you controlled for confounding variables in your analyses.*
Population characteristics	*Describe the covariate-relevant population characteristics of the human research participants (e.g. age, genotypic information, past and current diagnosis and treatment categories). If you filled out the behavioural & social sciences study design questions and have nothing to add here, write "See above."*
Recruitment	*Describe how participants were recruited. Outline any potential self-selection bias or other biases that may be present and how these are likely to impact results.*
Ethics oversight	*Identify the organization(s) that approved the study protocol.*

Note that full information on the approval of the study protocol must also be provided in the manuscript.

Field-specific reporting

Please select the one below that is the best fit for your research. If you are not sure, read the appropriate sections before making your selection.

☒ Life sciences ☐ Behavioural & social sciences ☐ Ecological, evolutionary & environmental sciences

For a reference copy of the document with all sections, see nature.com/documents/nr-reporting-summary-flat.pdf

Life sciences study design

All studies must disclose on these points even when the disclosure is negative.

Sample size	We did not use statistical methods to predetermine the sample size due to a lack of an effect size estimate. A total of 21 animals were included in the study (baseline drift: 6 mice, 28 day orientation deprivation: 7 mice, repeated 7 day orientation deprivation: 8 mice). Sample sizes were chosen based on recent studies.
Data exclusions	Two animals were excluded because their cylinder lens goggles fell off prematurely and the continuity of orientation deprivation could not be ensured.
Replication	Baseline preferred orientation drift was quantified across 6 mice. For the orientation deprivation experiment we quantified the preferred orientation drift and convergence across a total of 15 mice. We first observed preferred orientation convergence in a pilot experiment before confirming this in a a second larger cohort, which we report on in this manuscript.
Randomization	Animals were randomly assigned to either the repeated 7 day or the 28 day orientation deprivation experiment.
Blinding	The experimenters were not blinded to group allocation of mice, due to the different length of experiment. During analysis, blinding was not

Blinding	possible, due to the different number of imaging sessions between groups.

Reporting for specific materials, systems and methods

We require information from authors about some types of materials, experimental systems and methods used in many studies. Here, indicate whether each material, system or method listed is relevant to your study. If you are not sure if a list item applies to your research, read the appropriate section before selecting a response.

Materials & experimental systems

n/a	Involved in the study
☒	☐ Antibodies
☒	☐ Eukaryotic cell lines
☒	☐ Palaeontology and archaeology
☐	☒ Animals and other organisms
☒	☐ Clinical data
☒	☐ Dual use research of concern
☒	☐ Plants

Methods

n/a	Involved in the study
☒	☐ ChIP-seq
☒	☐ Flow cytometry
☒	☐ MRI-based neuroimaging

Animals and other research organisms

Policy information about studies involving animals; ARRIVE guidelines recommended for reporting animal research, and Sex and Gender in Research

Laboratory animals	For all experiments female wild-type C57BL/6NJr mice (Janvier) were used from the age of P35 (start of data acquisition ~P60). Mice were housed at 55 ± 5 % humidity and 22 ± 1.5°C under a 12-hour inversed light-dark cycle with food and water available ad libitum. Mice were housed in large cages (GR900, Tecniplast) with access to a running wheel and other enrichment material such as a tunnel and a house. Animals were usually group-housed, except for short time periods during recovery from surgery.
Wild animals	The study did not involve wild animals.
Reporting on sex	We only used female mice in order to ensure consistency of housing conditions.
Field-collected samples	The study did not involve samples collected from the field.
Ethics oversight	All experimental procedures were done according to institutional guidelines of the Max Planck Society and the regulations of the local government ethical committee (approved by Beratende Ethikkommission nach §15 Tierschutzgesetz, Regierung von Oberbayern).

Note that full information on the approval of the study protocol must also be provided in the manuscript.

Plants

Seed stocks	*Report on the source of all seed stocks or other plant material used. If applicable, state the seed stock centre and catalogue number. If plant specimens were collected from the field, describe the collection location, date and sampling procedures.*
Novel plant genotypes	*Describe the methods by which all novel plant genotypes were produced. This includes those generated by transgenic approaches, gene editing, chemical/radiation-based mutagenesis and hybridization. For transgenic lines, describe the transformation method, the number of independent lines analyzed and the generation upon which experiments were performed. For gene-edited lines, describe the editor used, the endogenous sequence targeted for editing, the targeting guide RNA sequence (if applicable) and how the editor was applied.*
Authentication	*Describe any authentication procedures for each seed stock used or novel genotype generated. Describe any experiments used to assess the effect of a mutation and, where applicable, how potential secondary effects (e.g. second site T-DNA insertions, mosiacism, off-target gene editing) were examined.*
